# Recompensation of Heart and Kidney Function after Treatment with Peritoneal Dialysis in a Case of Congestive Heart Failure

**DOI:** 10.1155/2011/197816

**Published:** 2011-11-17

**Authors:** Lars P. Kihm, Vinzent Hankel, Christian Zugck, Andrew Remppis, Vedat Schwenger

**Affiliations:** ^1^Department of Nephrology, University Hospital, Im Neuenheimer Feld 162, 69120 Heidelberg, Germany; ^2^Department of Cardiology, University Hospital, Im Neuenheimer Feld 410, 69120 Heidelberg, Germany; ^3^Department of Cardiology, Herz-Gefäßzentrum, Römstedter Straße 25, 29549 Bad Bevensen, Germany

## Abstract

We report the case of a 57-year-old woman suffering from congestive heart failure. Due to refractory congestions despite optimised medical treatment, the patient was listed for heart transplantation and peritoneal dialysis was initiated. Peritoneal dialysis led to a significant weight loss, reduction of hyperhydration and extracellular water obtained by bioimpedance measurement, and a significant improvement in clinical and echocardiographic examination. Furthermore, residual kidney function increased during the long-term followup, and subsequently peritoneal dialysis was ceased. Pulmonary artery pressure and left ventricular ejection fraction remained stable and the patient did well. This case demonstrates the possibility of treating hyperhydration due to congestive heart failure with peritoneal dialysis resulting in recompensation of both heart and kidney functions.

## 1. Background

Chronic congestive heart failure is an entity that may lead to progressive chronic kidney disease, then classified as cardiorenal syndrome type 2 [1]. The pathophysiology of renal dysfunction in the setting of advanced cardiac failure is not yet well understood; however, haemodynamic issues seem to play an important role, as well as renal congestion and neurohormonal abnormalities. A major problem in the treatment of cardiorenal syndrome type 2 is the progression of renal injury due to optimised drug therapies of cardiac failure, such as loop diuretics. The treatment of refractory congestive heart failure with peritoneal dialysis has been introduced in the late 40s [2] but has not yet become a standardized approach [3].

## 2. Case Report

This concerns a 57-year-old female patient, who introduced herself in 2002 for the first time in our outpatient clinic. At that point, an anterior myocardial infarction of unknown duration and a posterior myocardial infarction in 10/02 treated with double aortocoronary bypass were already known.

After accurate diagnostics, an ischaemic cardiomyopathy with a high-level constriction of the left ventricular ejection fraction (26% measured in TTE), a 3-vessel coronary artery disease, an insufficient mitral valve I°, an arterial hypertension, and hyperlipidaemia were recognised. As a result of triggerable ventricular extrasystoles, an AICD was implanted in 11/02.

In 7/2007, an infarction of the cerebri media artery led to a brachiofacial hemiparesis in the right territorial area with dysarthria and dysphagia on the left side. In 11/07, an ICD-CAT upgrade was accomplished.

In Spring 2008, in a status of repeated hydropic decompensation, the patient arrived again for admission to evaluate the possibility of a heart transplantation. After intensive and multidisciplinary discussion, the decision to wait-list the patient was reached despite the present comorbidities.

At this time, the cardiac capacity was very low with a maximum possible walking distance of 25 metres.

The clinical status worsened in June 2008 when there was a recrudescence of cardiac decompensation with hyperhydration, although maximal possible medical treatment was administered. The indication for dialysis had arisen, because of a repeated hydropic decompensation and a plainly reduced kidney function with a clearance of 26 mL/min/1.73 m (MDRD).

A Tenckhoff catheter was implanted without complications to start peritoneal dialysis in 06/08 with 9.5 L physioneal 1.36% and 1.5 L extraneal using automated peritoneal dialysis (APD).

**Figure 1:**
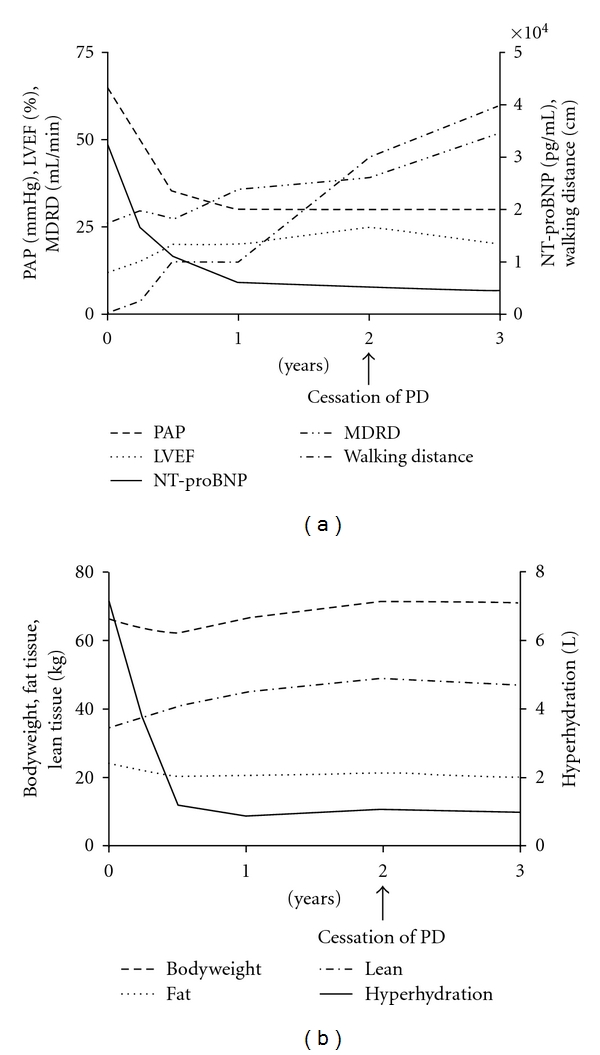
(a) Time course of pulmonary artery pressure (PAP), left ventricular ejection fraction (LVEF), and N-terminal probrain natriuretic peptide (NT-proBNP), residual renal function (MDRD), and walking distance after the initiation of peritoneal dialysis treatment. (b) Time course of bioimpedance measurement with the Body Composition Monitor regarding hyperhydration, bodyweight, lean tissue mass, and fat tissue mass after the initiation of peritoneal dialysis treatment.

As a clear result, the bodyweight was significantly reduced, including reaching consecutive cardiac recompensation. The bioimpedance measurement using the Body Composition Monitor system (Fresenius Medical Care, Bad Homburg, Germany) showed a rigorous reduction of hyperhydration and simultaneous increasing muscle mass and body weight as treatment proceeded. At the same time, the pulmonary artery pressure (PAP) was significantly reduced. The initial high-level NT-pro-BNP parameter decreased within the first weeks of dialysis as well as becoming stabilised during the whole treatment (see Figure 1).

An improved general condition and good acceptance of peritoneal dialysis were seen when the patient underwent the regular reassessments. The residual diuresis was quantified with 1.5 L with a Kt/V quality of 2.8 (1.4 renal and 1.4 peritoneal).

A successful APD treatment-free interval was initiated in 12/10, showing adequate diuresis and stable retention parameters leading to catheter removal in 03/11. Heart function was stable til 06/11, the calculated eGFR (MDRD) increased from 39.2 mL/min/1.73 m at the timepoint of PD cessation to 51.8 mL/min/1.73 m 06/11, and the possible walking distance increased up to kilometres walking the dog. Actually the patient is not listed for heart transplantation due to the clinical improvement.

## 3. Discussion

In this case, there was a cardiorenal syndrome type 2 including interference of advanced congestive heart failure with kidney function. The peritoneal dialysis treatment demonstrated its opportunity to impact on both heart and kidney. Despite comorbidities, heart transplantation was taken into account. After repeated hydropic decompensation despite optimised conservative medical treatment, there was certainly need for an alternative therapeutic approach. Peritoneal dialysis permitted not only the examination of capacity and cardiac recompensation, but also a recompensation of renal function. As this case shows, peritoneal dialysis offers a therapeutic option, at least in patients waiting for heart transplantation or for patients not possible for heart transplantation. The opportunity of an intermittent treatment with a subsequent abrogation of peritoneal dialysis treatment underlines the importance of fluid balance in these patients, which can be achieved by peritoneal dialysis and monitored with bioimpedance measurement.
